# Prevalence of hepatitis B virus co-infection among HIV-seropositive persons attending antiretroviral clinics in the Eastern Region of Ghana

**DOI:** 10.11604/pamj.supp.2016.25.1.6172

**Published:** 2016-10-01

**Authors:** Gideon Kye-Duodu, Priscillia Nortey, Keziah Malm, Kofi Mensah Nyarko, Samuel Oko Sackey, Sampson Ofori, Edwin Andrews Afari

**Affiliations:** 1 Ghana Field Epidemiology and Laboratory Training Programme (GFELTP), School of Public Health, University of Ghana, P.O. Box LG 13, Accra, Ghana; 2 Department of Epidemiology and Disease Control, School of Public Health, University of Ghana, P.O. Box LG 13, Accra, Ghana; 3 Ghana Health Service, Regional Hospital, Koforidua, Eastern Region, Ghana

**Keywords:** Human Immunodeficiency Virus, hepatitis B virus, co-infection, risk factors, Ghana

## Abstract

**Introduction:**

Hepatitis B and HIV infections are endemic in sub-Saharan Africa including Ghana. Understanding the extent of the co-infection is critical to the optimal care of persons living with HIV and AIDS (PLHIV). We determined the prevalence and risk factors of HBV co-infection in PLHIV and assessed the knowledge of health care workers (HCW) in Antiretroviral Therapy (ART) clinics regarding the co-infection.

**Methods:**

A cross sectional study was conducted in five ART clinics to obtain data from a systematic random sample of PLHIV in the Eastern region of Ghana from March to June 2012. We used self-administered questionnaires to assess knowledge of HCW on knowledge and management of the co-infection. Descriptive statistics and logistic regression models were used for analysis at 5% significance level.

**Results:**

Of 320 PLHIV recruited into study, with median age of 40 years (IQR: 33-50 years), 28 tested positive for HBsAg giving an overall prevalence of 8.8%. There were significant associations between HBV infection and being an adult (p=0.004), increasing serum ALT levels (p=0.002) and partner with history of HBV infection (p=0.010). HCW obtained 84.2% (SD± 20.53; 95% CI: 89-98.1) and 53.1% (SD± 35.06; 95% CI: 13.0-88.9) in the “general knowledge” and “management practice” indexes respectively.

**Conclusion:**

Prevalence of HBV-HIV co-infection was relatively high among PLHIV in Eastern region. Knowledge of HCW on management practices of HBV-HIV co-infection and HBV vaccination coverage among PLHIV were found to be relatively low. Regular trainings of HCW and a HBV vaccination programme targeted at PLHIV should be considered.

## Introduction

HBV and HIV have similar routes of transmission, hence co-infections are common which poses increased risk for life threatening complications for people living with both infections [1–3]. Studies of the co-infection have shown that HIV infection negatively impacts the natural history of hepatitis B leading to increased rates of persistent infection, loss of protective antibody against HBV, increased risk of liver cancer and liver-related mortality [1, 4]. In countries where highly active antiretroviral therapy (HAART) has been available for some time such as South Africa and Botswana [5–7], liver diseases associated with HBV, have emerged as a major cause of morbidity and mortality in persons living with HIV and AIDS (PLHIV) [8, 9]. The WHO in 2012 reported that more than 2 billion people have been infected with HBV at some time in their lives [10]. In 2010, the number of people globally living with HIV and AIDS was 34 million with 2.7 million new infections and 1.8 million AIDS-related deaths in that year alone [11]. Sub-Saharan Africa has only 12% of the global population, yet about 68% (approximately 22.5 million) of all people living with HIV reside in this region, as well as an estimated 50 million chronic HBV carriers [11].

Modes of transmission of hepatitis B are usually characterized by the geographical origin of infected patients. In areas with low HBV prevalence (less than 2%) such as North America and Western Europe, injecting drug use (IDU) and unprotected sex have been found to be the primary modes of transmission, and affect essentially the adult population [12]. In contrast, for high endemic areas (greater than 8%) such as Africa and Asia, close contact within households, medical or cultural procedures such as tattoos and scarification and perinatal transmission within the five years of life have been identified as the primary modes of transmission [13, 14]. A number of studies have examined the epidemiology of HIV and HBV individually in countries in sub-Saharan Africa, where both diseases have hit so hard, but few have studied HIV and HBV co-infection [12]. Kapembwa et al in 2011 estimated HBsAg seropositivity to be 9.9% of HIV-infected adults in Zambia [15]. Another study in Malawi among antenatal clients estimated a HBV-HIV co-infection prevalence of 17.5% [14]. In Ghana, a prevalence of 16.7% was found among a Ghanaian HIV-positive cohort hospitalized at a tertiary care institution in Kumasi [16]. The varying estimates obtained in different areas of sub-Saharan Africa clearly demonstrate the need for ongoing surveillance activities.

Ghana's HBV infection is estimated to be 15% of the adult population whereas the HIV prevalence in 2011 was 2.1%, a marginal rise from 2.0% in the previous year [17]. Since May 2003 when HAART was initiated in Ghana, there has been a massive expansion in care countrywide, with subsequent improvement in morbidity and mortality of PLHIV [18]. In response to new evidence, the National HIV/AIDS Prevention and Control Programme (NACP) revised the national ART guidelines and included for the first time, the management of hepatitis B and HIV co-infection. Though the HIV prevalence in the general population and that of hepatitis of B for different study populations of the infections are known, there is relatively less information to accurately establish the prevalence of the co-infection in Ghana [18]. Health care workers (HCW), who provide and monitor standard antiretroviral therapy to PLHIV are generally assumed to have adequate knowledge of the disease by virtue of their profession and proximity to the health facility but that may not always be the case [19, 20]. Their knowledge on hepatitis B and the management of the co-infection is critical in determining treatment outcomes. This paper estimates the prevalence of co-infection of HBV and HIV and its associated factors as well as the knowledge of health care workers on the management of this co-infection in selected ART clinics in the Eastern Region of Ghana.

## Methods

**Study design:** we conducted a multicentre cross-sectional study among Persons Living with HIV and healthcare workers in antiretroviral clinics in the Eastern region of Ghana from March to June 2012.

**Study area:** the Eastern region is one of ten regions in Ghana with a projected population of 2,420,927 and growth rate of 1.4% [21]. The region had consistently reported the highest HIV prevalence rates from 2003 to 2012 for both urban and rural sites in the annual HIV Sentinel Surveillance (HSS) [18, 22]. There were 30 hospitals, 58 health centres, 126 clinics and 315 functional Community-Based Health Planning and Services (CHPS) in the region. Of these, 20 had well established antiretroviral (ART) clinics at the time, 12 (60%) of which were in rural areas.

**Study population and sampling procedure:** for the purpose of this study, five ART clinics were selected based on the probability proportional to size sampling procedure [23]. The 20 functional ART clinics with their respective population sizes were listed to obtain a cumulative population of PLHIV in the Eastern region. This sampling procedure is described in detail elsewhere [24]. Using a sample size formula by Kish Leslie for cross-sectional studies [25], a sample size of 300 was obtained based on an estimated HBV-HIV co-infection prevalence of 17% found among a cohort of PLHIV in a tertiary hospital in Ghana [16] for a confidence interval of 95% and adjusting for a 15% non-response rate where there could be 5% drop out rate and a 10% inadequate quantity and/or contamination rate of blood serum. At each study site, PLHIV visiting the clinic were selected by a systematic random sampling procedure using a box draw, where a written number from one to nine, on a piece of paper was picked from a box each morning to obtain a sampling interval. All enrolled PLHIV were interviewed using structured questionnaires to collect information on demographic characteristics, medical history, knowledge about HBV-HIV co-infection, socio-economic status and risk factors for hepatitis B infection. HIV counselors, known by most of the participants were trained to conduct interviews in counselling rooms. A total of fifty-four health workers available in the ART sites within the period were also recruited and interviewed for the study. The selected ART clinics were Asesewa Government Hospital, Atua Government Hospital and Begoro Government Hospital in rural locations and St. Dominic's Hospital in Akwatia and the Regional Hospital in Koforidua, both in urban areas.

**Inclusion criteria for recruiting PLHIV:** any ART client ≥ 2 years reporting to any of the selected clinics was eligible to participate irrespective of previous testing for hepatitis B. Informed consent and assent from children was obtained from each eligible client and/or parent of children.

**Exclusion criteria for recruiting PLHIV:** Failure to obtain informed consent, children less than 2 years old who had not tested for HIV (PCR DNA) and persons who were too ill to undertake the procedures involved in the study were excluded from the study.

**Health care workers:** fifty-four health care workers that were available in the selected ART clinics during the period of study were recruited to complete a self-administered 22-question survey derived from the ART guidelines, to ascertain their knowledge of hepatitis B/HIV co-infection and its management.

**Laboratory investigations and records review:** blood specimen (8 ml for adults and 5ml for children) were collected aseptically into 10 ml vacutainer tubes (Becton & Dickinson, NJ USA) and centrifuged within 30 minutes to obtain serum for serological assays. The specimen were kept frozen at the study sites for not more than two weeks and transported under cold chain (2-80C) to the NPHRL and Virology Department of the University of Ghana Medical School (UGMS) for serological investigations. Samples that tested HBV positive (measured as HBsAg positive) were further tested for hepatitis B serologic markers; hepatitis B core antigen (HBcAb), hepatitis B e antibody (HBeAb) and hepatitis B e antigen (HBeAg) with ELISA kits (Foresight, Acon Laboratories, Inc., USA). The most current clinical data for each participant, including CD4+ T-lymphocyte counts, haemoglobin concentration (HB), alanine aminotransferase (ALT) and aspartate aminotransferase (AST) levels was extracted from the folders of study participants, where available.

**Data processing and analysis:** data was expressed as frequencies and percentages to estimate the overall prevalence. Bivariate analyses were done to assess the associations between HBV infection and socio-demographic characteristics of study participants at corresponding 95% confidence interval. Pearson's chi-square test was used to compare categorical variables where appropriate. A p -value of <0·05 was considered significant. Factors associated with HBV infection that were significant in the bivariate analyses were put into the logistic regression model. We measured the knowledge levels of HCW on HBV-HIV co-infection by grouping the questions into two sections; HBV and HIV general knowledge and HBV-HIV management practices. The maximum possible score for each question was 100% and the minimum possible score was 0%. The questions in each section were then used to compile a “general knowledge” and a “knowledge of management practice” index respectively. Each index was then calculated by finding the average of the score of questions in each section. Each index was graded as follows: Poor Knowledge = 0%-50%; Fair Knowledge = 51%-70%; Good Knowledge = 71%-100% Data was entered into Epi Data version 3.1 software and analysed in SPSS 16.

**Ethical considerations:** ethical clearance was obtained from the Research and Ethical Review Committee of the Ghana Health Service and prior permission sought from the managers of selected health facilities. Study participants were fully informed about the purpose, procedures, risks and benefits of participation. Voluntary informed consent was sought from adults and parents or guardians of children and assent from older children prior to enrolment. Responses were kept confidential and data collected was kept for the purpose of this study only.

## Results

### Socio-demographic Characteristics of PLHIV

Three hundred and twenty PLHIV were recruited in all, 232 (72.5%) of whom were females. Their ages ranged from 4 years to 76 years with a median age of 40.0 years (IQR: 33-50 years). Males were significantly older, with mean age of 43.9 years (±14.3) compared to females with mean age of 39.17 years (±11.7), p=0.01. Table 1 summarizes the socio-demographic characteristics of study participants. Twenty eight PLHIV tested positive for HBV, giving an overall HBV-HIV co-infection seroprevalence of 8.8% [95% C.I: 6%-12.5%]. PLHIV aged 31 - 40 years reported the highest co-infection prevalence of 4.4% (14/320), whiles none was observed in the 21 - 30 years age group. Children (less than 18 years) were more infected 30.7% (4/13) compared to adults (7.8%, 24/307), p=0.004. Of the 28 HBV positive participants, 26 (93%) had antibodies to hepatitis B core antigen (HBcAb). Hepatitis B e antigen (HBeAg) was present in nine (32.1%) of participants, whereas 10 (35.7%) had antibodies to the hepatitis B e antigen (HBeAb). Study participants from rural areas constituted 58.8% (188/320) of study population, of which 7.9% (15/188; p=0.560) were co-infected.

**Table 1:** Socio-demographic characteristics of PLHIV (n=320)

Characteristic	Male (%)	Female (%)	Total (%)	p value
	n=88	n=232	n=320	
**Age Group**				0.000
2-10	4 (1.3)	2 (0.6)	6 (1.9)	
11-20	3 (0.9)	6 (1.9)	9 (2.8)	
21-30	4 (1.3)	36 (11.3)	40 (12.5)	
31-40	19 (5.9)	100 (31.3)	119 (37.2)	
41-50	27 (8.4)	50 (15.6)	77 (24.1)	
51+	31 (9.6)	38 (11.8)	68 (5.3)	
**Ethnicity**				0.874
Akyem	14 (4.4)	29 (9.1)	43 (13.4)	
Krobo	42 (13.1)	111 (34.8)	153 (47.8)	
Others	36 (11.3)	88 (27.5)	124 (38.8)	
**Education**				0.070
None	13 (4.1)	71 (22.2)	84 (26.3)	
Primary	43 (13.4)	113 (35.3)	156 (48.8)	
Secondary	33 (10.3)	41 (12.8)	74 (23.1)	
Tertiary	3 (0.9)	3 (0.9)	6 (1.9)	
**Occupation**				0.416
Trading/Business	29 (9.1)	141 (44.1)	170 (53.1)	
Farmer	46 (14.4)	54 (16.9)	100 (31.2)	
Others	17 (5.3)	33 (10.3)	50 (15.6)	
**Income Level**				0.519
<GHC100	61 (19.1)	192 (60)	253 (79.1)	
GHC101-GHC250	19 (6.0)	30 (9.4)	49 (15.3)	
GHC251-GHC500	7 (2.2)	4 (1.3)	11 (3.4)	
>GHC500	5 (1.6)	2 (0.6)	7 (2.2)	
**Payment of HAART Services**				0.601
Out-of-pocket	58 (18.1)	136	194 (60.1)	
Employer	13 (4.1)	24	37 (11.6)	
Relatives	1 (0.3)	0 (0)	1 (0.3)	
NHIS	13 (4.1)	50 (15.6)	63 (19.7)	
PLHIV Group	4 (1.3)	11 (3.4)	15 (4.7)	
Other	3 90.9)	7 (2.2)	10 (3.1)	

**Figure 1:**
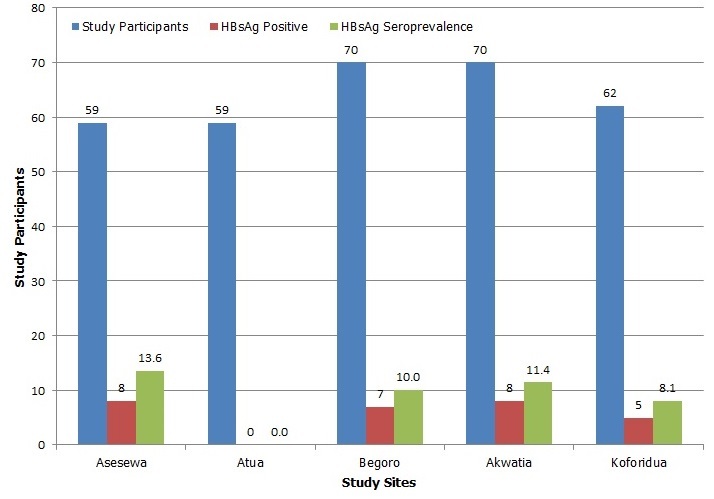
HIV-HBV seroprevalence in ART clinics in Eastern region of Ghana, 2012

The proportion of HBV-HIV co-infected individuals varied across ART study sites, with a highest of 13.6% (Figure 1). The mean CD4 count and haemoglobin concentration of co-infected individuals were 467 cells/µl and 12.9 g/dl respectively. Mean counts for biochemical markers, ALT and AST were also 36.07 U/l and 40.53 U/l respectively (Table 2). There was a statistically significant association with elevated serum ALT levels (>54 U/L) and hepatitis B co-infection, p=0.002. Factors associated with hepatitis B infection such as lack of knowledge about hepatitis B infection, no previous testing for HBV and vaccination against HBV, scarifications marks or tattoos on body were identified. Four out of 311 (1.3%) reported to have vaccinated against HBV, out of which only 2 (50%) completed the full course. Of 309 study participants, 49 (15.9%) reported intravenous drug use (IDU) and 48/311 (15.4%) reported or had scarification marks and/or tattoos on their bodies.

**Table 2:** Characteristics of PLHIV and hepatitis B co-infection, Eastern region, 2012 (N=320)

Characteristics	HBsAg+(n=28)	HBsAg-(n=292)	p value
**Age Group**			0.476
2-10	1	5	
11-20	3	6	
21-30	0	41	
31-40	14	101	
41-50	6	71	
51+	4	68	
**Sex**			0.678
Male	9	83	
Female	19	213	
**Age Category**			**0.004**
Adult (≥18years)	24	283	
Children	4	9	
**Study Site**			0.560
Rural	15	173	
Urban	13	119	
**Education**			0.083
None-Primary	23	217	
Secondary	4	70	
Tertiary	1	5	
**Marital Status**			0.582
Single	9	38	
Married	9	132	
Divorced/Widowed	8	98	
Co-habiting	1	12	
Unknown	0	13	
**Clinical Data**			
Mean CD4+ count (cells/µl)	363.2	476.2	0.283
Mean ALT (U/L)	39.3	35.8	**0.002**
Mean AST (U/L)	44.9	40.2	0.061

### Knowledge of HBV infection by PLHIV

Knowledge about partner's hepatitis B positive status prior to the study (p=0.01, aOR=7.968) was found to be significantly associated with HBV infection (Table 3). Of six study participants who reported testing positive for HBV in the past, only three (50%) retested positive (ELISA), and three (9.1%) out of 33 who reported testing negative previously for hepatitis B tested positive with ELISA. These two variables were reported in different subjects and found to be mutually exclusive (p=0.020).

**Table 3:** Risk factors of hepatitis B infection among PLHIV, Eastern region, 2012

Risk factor	aOR (95% CI)	p value
Heard about hepatitis B	0.778 (0.36-1.72)	0.550
HBV positive partner	7.968 (1.26-50.3)	0.010
Had vaccination for hepatitis B	3.538 (0.355-35.24)	0.251
Intravenous drug use	0.678 (0.19-2.35)	0.539
Had blood transfusions	1.217 (0.46-3.16)	0.686
Scarification marks/tattoos	1.030 (0.33-2.62)	0.958

### Health care workers

We also recruited a total of 54 HCW made up of 26 (51.9%) nurses, 11 (20.4%) laboratory technicians, 6 (11.1%) medical officers, 3 (9.3%) pharmacists and 4 (7.4%) biostatisticians and 4 (7.4%) volunteers. Most of them, 24 (44.4%) were aged 20 years -59 years. The median years of experience working at the ART clinic was 3.0 years ranging from 1 - 29 years.

### General knowledge of hepatitis B among HCW

Though most HCWs in the study sites, 98.1% (53/54) had heard about HBV infection and also knew that a person could be infected with both HBV and HIV, 51.8%(28/54) thought that HBV infection was just another name for liver disease. About 61.1% (33/54) HCW did not think that HBV could be transmitted as a hospital-acquired infection. The mean score for general knowledge of hepatitis B infection among the HCW in this study was 84.2% (SD± 20.53; 95% CI: 89-98.1)

### Knowledge of HBV-HIV management among HCW

Most HCW, 94.4% knew that screening all PLHIV for HBV was part of baseline tests but only 50% and 48% of them knew the right time to initiate HAART in adults and children respectively. About 61% knew that a positive HBV repeat test indicated chronic infection and only 48.1% knew when to monitor a PLHIV client with HBV-HIV co-infection. Only 13% of study participants knew about the existence of other hepatitis B serologic markers besides HBsAg. Study participants obtained a mean score for “management practice” index of 53.1% (SD ±35.06; 95% CI: 13.0-88.9).

## Discussion

We estimated the prevalence of HIV-HBV co-infection and its risk factors as well as the knowledge of HCW on its management in the Eastern region of Ghana. We found HBV seroprevalence (measured as HBsAg seropositivity) of 8.8% among PLHIV attending antiretroviral clinics in rural and urban sites in the Eastern region, consistent with Puoti et al.'s worldwide estimate of 5%-10% HBV-HIV co-infection prevalence [3]. For intermediate to high HBV endemic countries, HBV-HIV co-infection prevalence rates have been found to be 10-20% [13, 14]. For an estimated 15% hepatitis B prevalence, Ghana falls into WHO's bracket of hepatitis B endemic countries and our estimate is only slightly lower. Similar co-infection estimates were reported in Zambia, 9.9% [15], Tanzania, 17.3% [26] and Botswana, 10.6% [27]. However, our estimate was lower than other studies in Ghana by Geretti et al in 2008 that found 16.7% HBV-HIV co-infection seroprevalence rate [16] and Blankson et al, 2005 (42.5%) [28], both of which were conducted in tertiary hospitals. These hospitals are referral centers and this might have contributed to the high prevalence that was found. For similar outpatient settings, our estimate was higher than that found in Ethiopia [29] and Malawi [30] two countries with relatively higher HIV prevalence rates and longer HAART experiences compared to Ghana.

The varying estimates show the difference in risk factors in these populations. One study in Brazil reported more than 50% intravenous drug use among study participants with HCV/HIV [31], whiles another in Nigeria found that 50% of study participants with scarification marks and having unprotected sex were HIV and viral hepatitis co-infected [32]. The highest co-infection prevalence was found in the ages 31 years to 50 years, similar to that found in Pereira's study (2006) in the state of Mato Grosso, Brazil [33]. Acute hepatitis B infections often resolve spontaneously, especially in adults, but HIV infection has been found to negatively impact on the natural course of hepatitis B infection leading to loss of protective antibody against HBV. This could be the likely experience for these individuals, who may have had prior exposure to hepatitis B. Hepatitis B and HIV co-infection prevalence among rural and urban populations have shown marked differences in Africa. In Turkey, 37% of hepatitis B infection was found in rural areas compared to 67% in urban areas [34]. Our study found a contrasting high prevalence of 13.6% in a rural site compared to the highest urban prevalence of 11.4%, though there was no significant association (p=0.560; OR=0.794 rural/urban). One of the major determinants of hepatitis B infection is the age of acquiring the infection [12]. In this study, we observed a significant association between co-infection and being an adult (p=0.004), which is a widely observed fact, due to increasing risk of exposure with time. Similar findings were observed by Pereira (2006) in Brazil [33] and Nagu in Tanzania [26].

In this study, classic risk factors for viral hepatitis infection such as intravenous drug use, scarification marks and/or tattoos on body and lack of hepatitis vaccination were not associated with hepatitis B infection. Though IDU is a growing risk factor for HIV and hepatitis in sub-Saharan Africa, especially among the youth [35], our study participants were relatively older and predominantly farmers and traders. This is not a typical profile of people who use drug intravenously. On the other hand, scarification marks are commonly found among some ethnic groups in Northern Ghana [36], possibly explaining why a small number of our study participants reported these factors. Having tested for HBV prior to the study or a partner doing so was significantly associated to hepatitis infection and the most likely postulate could be that they might had shown signs and symptoms of the infection prompting a test to diagnosis it.

To reduce the incidence of hepatitis B infection in PLHIV, the guidelines for ART in Ghana, like many others, recommend HBsAg baseline test and vaccination for PLHIV. Despite this, only 1.3% of those interviewed reported previous vaccination against HBV. A number of studies in sub-Saharan Africa also observed low HBV vaccination coverage for PLHIV and even for HCW [37]. The NACP provides hepatitis B test kits to ART clinics but the onus is on PLHIV to get their own vaccinations to prevent them from life threatening complications of co-infection. Of the 28 HBsAg positive participants, 26 (93%) had antibodies to hepatitis B core antigen (HBcAb) signifying past or current infection. Combined with a positive test for HBsAg usually indicates a chronic infection. HIV can negatively impact on the natural course of HBV leading to the loss of protective antibody against HBV and this might explain the high percentage of study participants who tested positive for both markers. Hepatitis B e antigen (HBeAg) was present in nine (32.1%) of participants; signifying active viral replication whereas 10 (35.7%) had antibodies to the hepatitis B e antigen (HBeAb) signifying HBeAg seroconversion or end-point for treatment.

Our study found that 84.2% of ART health care workers had the right knowledge of HBV infection. Surprisingly, 11.1% of them thought that shaking hands with an infected person could transmit HBV and 9.3% did not know that sexual intercourse was a possible route of transmission for HBV. A health care provider with this kind of knowledge may be unwilling to touch hepatitis B infected individuals, depriving them of the needed care. It is believed that knowledge is usually the first step towards the modification of a desirable behaviour and it is therefore imperative for all HCWs to have adequate knowledge in this area to help them to provide the right management of HBV co-infection in PLHIV. Again, HCW are expected to educate and counsel their clients with the correct preventive and disease transmission information and they can only do that effectively if they are better informed themselves. The low “management practice” index in this study may be due to the fact that the ART guidelines are relatively new and time is needed for HCW to be adequately trained on them.

### Limitations

Only the 28 HBsAg positive participants were further screened for hepatitis B serologic markers. HBsAg testing was only done once, making it impossible to characterize the HBV infection as chronic, based on definition of persistent HBsAg+ for a minimum of six months. Some of the HBsAg positive could have had acute infection that subsequently cleared. Testing all study participants for HBcAb (antibodies to hepatitis B core Antigen) could have detected the presence of persistent, low-level carriers and those in the window period of acute infection. Testing for HBc IgM could have also detected PLHIV with occult HBV infection (HBc IgM positive but HBsAg negative). The risk factors and results of previous testing for hepatitis B were mostly self-reported and could be subjective and influenced by recall bias. We controlled for this weakness to an extent by asking participants to show evidence of their test results, especially for those who had the test in the same hospital.

## Conclusion

Our study showed that hepatitis B infection among PLHIV attending antiretroviral clinics in the Eastern region of Ghana is slightly lower than worldwide estimates of the co-infection for intermediate to high HBV endemic countries, though relatively higher than some other regions in sub-Saharan Africa with similar settings. HBV vaccination was low among PLHIV as well as HCW knowledge of the management of the co-infection. Training of health care workers, especially on the new ART guidelines and a hepatitis B vaccination programme targeted at PLHIV should be considered.
